# Relationship between intraventricular hemorrhage and acute kidney injury in premature infants and its effect on neonatal mortality

**DOI:** 10.1038/s41598-021-92746-3

**Published:** 2021-06-24

**Authors:** Mountasser M. Al-Mouqdad, Roya Huseynova, Thanaa M. Khalil, Yasmeen S. Asfour, Suzan S. Asfour

**Affiliations:** 1 Neonatal Intensive Care, Hospital of Paediatrics, King Saud Medical City, Al Imam Abdul Aziz Ibn Muhammad Ibn Saud, Riyadh, 12746 Saudi Arabia; 2 Obstetric and Gynecology Department, Maternity Hospital, King Saud Medical City, Riyadh, Saudi Arabia; 3 Obstetric and Gynecology Department, Family Care Hospital, Riyadh, Saudi Arabia; 4 Clinical Pharmacy Department, Pharmaceutical Care Services, King Saud Medical City, Riyadh, Saudi Arabia

**Keywords:** Medical research, Nephrology, Risk factors

## Abstract

Intraventricular hemorrhage (IVH) and acute kidney injury (AKI) are important neonatal morbidities in premature infants. This study aimed to investigate the relationship between IVH and AKI in premature infants and whether this association affects the incidence of neonatal mortality. Infants [gestational age (GA) ≤ 32 weeks; birth weight (BW) < 1500 g] were retrospectively evaluated in a large tertiary neonatal intensive care unit. Of 710 premature infants, 268 (37.7%) developed AKI. Infants with IVH were more likely to have AKI than those without IVH. Infants with severe IVH had a higher incidence of AKI than infants with mild IVH. Infants younger than 28 weeks with IVH were more likely to have AKI than those without IVH. An association between IVH grades and AKI stages was observed in the overall study population, in infants with GA < 28 weeks, and in infants with GA between 28 and 32 weeks. Mortality was increased 1.5 times in infants with IVH and AKI compared with that in infants with IVH but without AKI. Furthermore, mortality was increased in infants with IVH and AKI compared with infants without IVH or AKI. This study shows a direct relationship between the severity of IVH and the degree of AKI; both IVH and AKI increase the incidence of neonatal mortality.

## Introduction

Despite many advances in neonatal care that improve the survival rate of premature infants, the incidence of major neonatal morbidities remains significantly unchanged. Intraventricular hemorrhage (IVH) is the most common short-term neurological manifestation and remains a major problem of prematurity^1,2^. As the survival rate of premature infants increases, the incidence of IVH becomes more prominent. Further, it aggravates the risk of death and short- and long-term neurodevelopmental deficits^3,4^.

The kidneys play a crucial role in regulating blood pressure through the renin–aldosterone–angiotensin system (RAAS)^5^. Acute kidney injury (AKI) is a complication observed in neonates, and recently, many studies have demonstrated the importance of AKI and its influence on neonatal outcomes especially in premature infants. Moreover, AKI plays a critical role in the development of neonatal hypertension, chronic lung disease, and chronic kidney injury^6–9^; infants with AKI stay longer in the hospital and are at high risk of mortality^10^.

Most of the studies that investigated the short-term complications of IVH focused mainly on the development of posthemorrhagic hydrocephalus and the extension of bleeding into the white matter, thus causing additional injury^11,12^. However, these studies did not focus on the influence of IVH on other systemic conditions, such as the renal system. IVH is known to cause systemic hypotension and acidosis, which can affect renal perfusion and cause renal impairment. On the other hand, any disturbance in renal function would contribute to the development of IVH through either dysregulating the RAAS or attenuating the inflammatory system^5,13^.

To date, only a few studies have focused on the relationship between AKI and IVH; these studies assumed that infants with AKI are at high risk of developing IVH^14,15^. However, in the present study, we aimed to elucidate the association between AKI and IVH in a new population. We hypothesized that IVH is a risk factor for developing AKI and their combination increased the risk of neonatal mortality.

## Results

**Figure 1:**
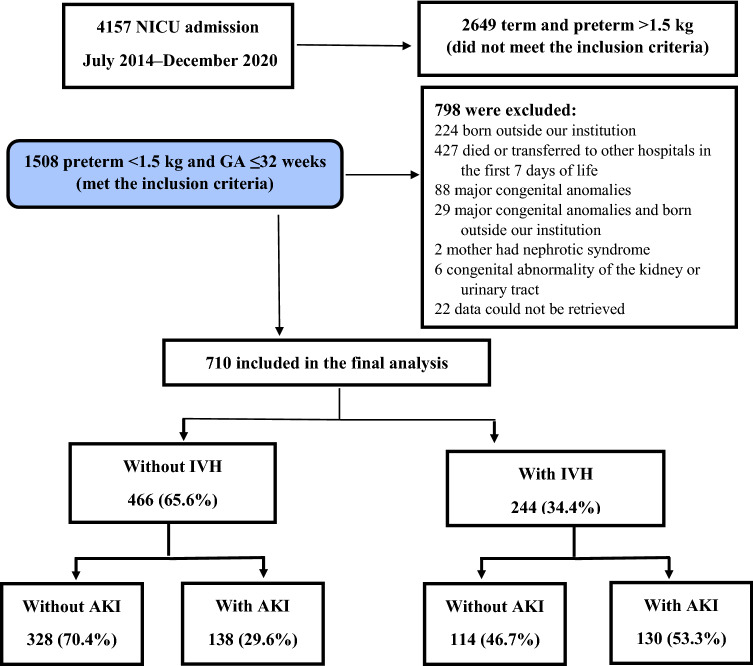
Flow chart of patient selection. Abbreviations: *GA* gestational age, *NICU* neonatal intensive care unit, *IVH* intraventricular hemorrhage, *AKI* acute kidney injury.

During the study period, of 1508 preterm infants with a gestational age (GA) ≤ 32 weeks and a birth weight (BW) > 1500 g who were admitted to the neonatal intensive care unit (NICU, level 3), 710 met the inclusion criteria and were eligible for inclusion in the final analysis (Fig. 1). Seven hundred and ten infants were divided into four groups: IVH with AKI (n = 130), IVH without AKI (n = 114), without IVH or AKI (n = 328) and AKI without IVH (n = 138). The demographic characteristics of the mothers and infants stratified according to the presence of IVH and AKI are presented in Table 1.

**Table 1:** Comparison of demographic, clinical, and maternal characteristics among infants with and without IVH and/or AKI (n = 710).

Parameters	IVH with AKI (n = 130)	IVH without AKI (n = 114)	No IVH with No AKI (n = 328)	No IVH with AKI (n = 138)	(*P* value)^b^	(*P* value)^c^
Gestational age, weeks	27 (25–29)	27 (26–29)	30 (28–31)	30 (27–31)	0.04*	< 0.001*
Birth weight, grams	870 (695–1082.5)	965 (798.75–1221.25)	1230 (1020–1400)	1213.5 (927.5–1430)	0.003*	< 0.001*
Booked	19 (14.6)	17 (14.9)	39 (11.9)	29 (21)	> 0.99	0.43
Antenatal steroid treatment	59 (45.4)	58 (50.9)	182 (55.5)	73 (52.9)	0.44	0.06
Cesarean section delivery	63 (48.5)	46 (40.4)	142 (43.3)	58 (42)	0.24	0.34
Gestational diabetes mellitus	4 (3.1)	4 (3.5)	23 (7)	4 (2.9)	> 0.99	0.12
Maternal hypertension	22 (16.9)	25 (21.9)	92 (28)	63 (26.1)	0.334	0.01*
1-min Apgar score	4 (2–6)	5 (3–6)	6 (4–7)	6 (4–7)	0.09	< 0.001*
5-min Apgar score	6 (5–7)	7 (6–7)	7 (7–8)	7 (6–8)	0.03*	< 0.001*
RDS surfactant use	111 (85.4)	88 (77.2)	167 (50.9)	69 (50)	0.13	< 0.001*
Male sex	70 (53.8)	65 (57)	164 (50)	72 (52.2)	0.69	0.47
Non-invasive ventilator	88 (67.7)	95 (83.3)	299 (91.4)	120 (87)	0.005*	< 0.001*
Invasive ventilator	115 (88.5)	101 (88.6)	187 (57)	80 (58)	> 0.99	< 0.001*
PDA medical treatment	17 (13.1)	15 (13.2)	17 (5.2)	11 (8)	> 0.99	0.006*
Pneumothorax	18 (13.8)	9 (7.9)	8 (2.4)	6 (4.3)	0.15	< 0.001*
Pulmonary hemorrhage	22 (16.9)	9 (7.9)	5 (1.5)	5 (3.6)	0.05	< 0.001*
EBM	58 (44.6)	59 (51.8)	159 (48.5)	68 (49.3)	0.3	0.46
Inotropes	96 (73.8)	61 (53.5)	74 (22.6)	40 (29)	0.001*	< 0.001*
UAC	61 (46.9)	51 (44.7)	89 (27.1)	22 (15.9)	0.79	< 0.001*
UVC	113 (86.9)	98 (86)	244 (74.4)	87 (63)	0.85	0.004*
EOS	2 (1.8)	4 (4)	3 (1.1)	3 (2.4)	0.42	0.63
LOS	49 (43.4)	35 (35.4)	61 (22.7)	28 (22.4)	0.26	< 0.001*
Nephrotoxic medication	121 (93.1)	100 (87.7)	290 (88.4)	123 (89.1)	0.18	0.17
LOHS	44 (26–74)	55 (27–87)	36 (21–55.75)	36.5 (21.5–56.25)	0.38	< 0.001*
Mortality	57 (43.8)	24 (21.1)	25 (7.6)	22 (15.9)	< 0.001*	< 0.001*

Data are presented as median (IQR), or number (%), as appropriate.

Abbreviations: *IVH* intraventricular hemorrhage, *AKI* acute kidney injury, *GDM* gestational diabetes mellitus, *RDS* respiratory distress syndrome, *PDA* patent ductus arteriosus, *EBM* expressed breast milk, *UAC* umbilical arterial catheter, *UVC* umbilical venous catheter, *EOS* early-onset sepsis, *LOS* late-onset sepsis, *LOHS* length of hospital stay.

*Statistically significant at 5% level.

Bold values indicate all values less than 0.05 are significant.

^a^Nephrotoxic medication: Acyclovir, Amphotericin B, Aminoglycosides, Vancomycin.

^b^Comparison between IVH with AKI versus IVH without AKI.

^c^Comparison between IVH with AKI versus no IVH with no AKI.

Infants with AKI and IVH had a significantly lower GA, BW, and 5-min Apgar score than infants with IVH but without AKI. Furthermore, infants with AKI and IVH required more inotropes and had a high mortality rate (Table 1).

Comparison of infants with IVH and AKI to those without IVH or AKI revealed a significant association in most variables. Infants with IVH and AKI had a significantly lower BW, GA, and 1- and 5-min Apgar scores (*P* < 0.001). Further, those with IVH and AKI were less likely to be born to mothers who had hypertension (*P* = 0.01) (Table 1).

In regard to clinical course, infants with IVH and AKI were more likely to have umbilical arterial and venous catheters (UAC and UVC, respectively), receive surfactant, require inotropes, and be on a mechanical ventilator compared with those without IVH or AKI (*P* < 0.001, *P* = 0.004, *P* < 0.001, *P* < 0.001, and *P* < 0.001, respectively). Furthermore, they had higher rates of pneumothorax, pulmonary hemorrhage, and late-onset sepsis (*P* < 0.001) as well as a lower survival rate than infants without IVH or AKI (*P* < 0.001) (Table 1).

The association between AKI stages and IVH grades shows that a high percentage (46.2%) of infants in the cohort had neither IVH nor AKI. Infants with IVH were more likely to have AKI than those without IVH [53.3% (130/244) vs. 29.6% (138/466), *P* < 0.0001] (Table 2).

**Table 2:** Association between AKI stages and IVH grades (n = 710).

≤ 32 weeks (n = 710)						< 28 weeks (n = 235)				28–32 weeks (n = 475)			
	No IVH (n = 466)		IVH (n = 244)			No IVH (n = 97)		IVH (n = 138)		No IVH (n = 369)		IVH (n = 106)	
AKI stage	AKI (n = 268)	(*P* value)	Mild (n = 125)	Severe IVH (n = 119)	(*P* value)	AKI (n = 115)	(*P* value)	Mild (n = 53)	Severe (n = 85)	AKI (n = 153)	(*P* value)	Mild (n = 72)	Severe IVH (n = 34)
None	328 (70.4)	< 0.001*	69 (55.2)	45 (37.8)	0.03*	62 (63.9)	0.01*	25 (47.2)	33 (38.8)	266 (72.1)	< 0.001*	44 (61.1)	12 (35.3)
Stage 1	61 (13.1)		34 (27.2)	39 (32.8)		20 (20.6)		17 (32.1)	28 (32.9)	41 (11.1)		17 (23.6)	11 (32.4)
Stage 2	49 (10.5)		15 (12)	22 (18.5)		11 (11.3)		9 (17)	17 (20)	38 (10.3)		6 (8.3)	5 (14.7)
Stage 3	28 (6)		7 (5.6)	13 (10.9)		4 (4.1)		2 (3.8)	7 (8.2)	24 (6.5)		5 (6.9)	6 (17.6)

Abbreviations: *IVH* intraventricular hemorrhage, *AKI* acute kidney injury.

*Statistically significant at 5% level.

Bold values indicate all values less than 0.05 are significant.

Among those who did have IVH and AKI, those with mild IVH had the greatest number of neonate stage 1 AKI followed by stage 2 AKI and later stage 3 AKI. Similarly, infants with severe IVH also had the highest number of neonate stage 1 AKI followed by stage 2 AKI and later stage 3 AKI. Interestingly, infants with severe IVH had a higher incidence of AKI (stages 1–3) compared with infants with mild IVH (Table 2).

Infants younger than 28 weeks with IVH were more likely to have AKI than those without IVH [58% (82/138) vs. 36.1% (35/97), *P* = 0.01]. However, no relationship could be demonstrated when comparing mild IVH with severe IVH (*P* = 0.63) (Table 2).

Furthermore, infants at 28–32 weeks GA with IVH were more likely to have AKI than those without IVH [39.1% (60/106) vs. 27.9% (103/369), *P* < 0.001]. However, there was no significant difference between infants with mild IVH and those with severe IVH (*P* = 0.07) (Table 2).

The results of the ordinal logistic model for the overall study population suggested that infants with IVH were 2.3 times more likely to have AKI than infants without IVH (Table 3). This association remained significant after adjusting for GA, BW, 1- and 5-min Apgar scores, pulmonary hemorrhage, and pneumothorax (Table 3).

**Table 3:** Multivariable ordinal logistic regression for the association between IVH grades and AKI stages; overall and stratified by GA (n = 710).

AKI stage	Crude OR	CI	(*P* value)	Adjusted OR	CI	(*P* value)
Overall ≤ 32 weeks	2.3	1.69–3.12	< 0.001*	1.92	1.34–2.76	< 0.001*
≤ 8 27weeks	2.26	1.36–3.77	0.002*	1.94	1.11–3.39	0.02*
28–32 weeks	2	1.3–3.07	0.001*	2.03	1.29–3.2	0.002*

Abbreviations: *IVH* intraventricular hemorrhage, *AKI* acute kidney injury, *GA* gestational age, *OR* odds ratio, *CI* confidence interval.

*Statistically significant at 5% level.

Bold values indicate all values less than 0.05 are significant.

Additionally, the association between IVH grades and AKI stages was significant for infants with a GA < 28 weeks and a GA between 28 and 32 weeks. The adjusted association between IVH grades and AKI stages remained significant for infants with a GA < 28 weeks after controlling for confounder variables, including GA, BW, 1- and 5-min Apgar scores, and use of an invasive ventilator (Table 3).

**Figure 2:**
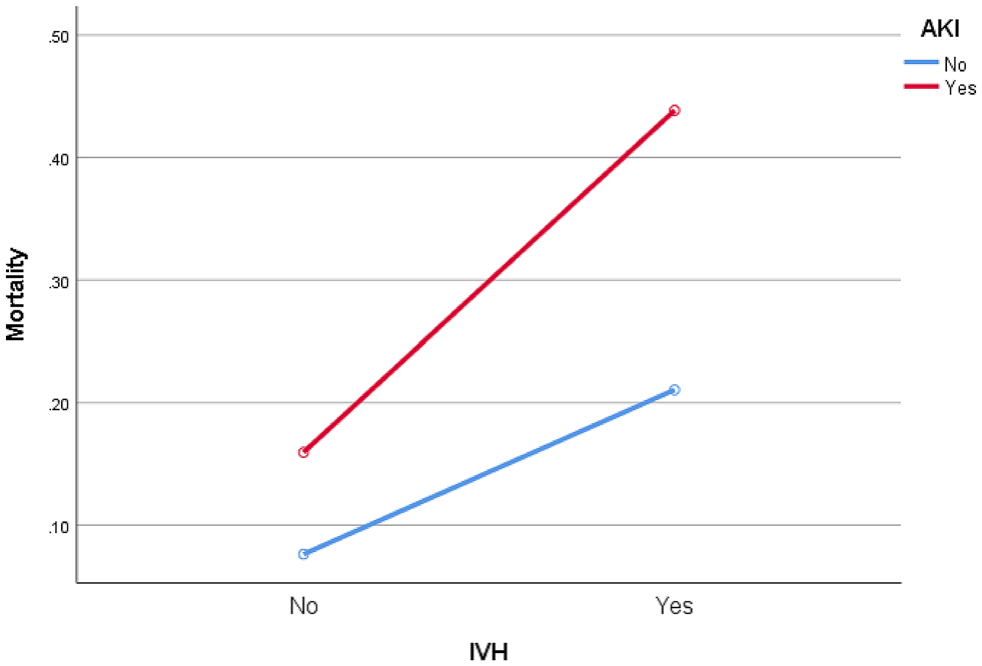
Interaction between IVH and AKI and the estimated marginal means of neonatal mortality (n = 710). Abbreviations: *IVH* intraventricular hemorrhage, *AKI* acute kidney injury.

An association between IVH grades and AKI stages also remained significant for infants with a GA between 28 and 32 weeks after controlling for confounder variables, including pulmonary hemorrhage and UAC and UVC insertion (Table 3). The interaction model indicated that AKI in premature infants with IVH had the strongest association with neonatal mortality (*P* = 0.015) (Fig. 2).

We further ensured that the GA and BW did not affect the primary outcome by performing 1:1 matching for a group of infants with IVH and AKI versus infants with IVH but without AKI and infants with IVH and AKI versus infants without IVH or AKI. We found that mortality increased in infants with IVH and AKI compared with infants with IVH but without AKI (*P* = 0.01). Mortality also increased in infants with IVH and AKI compared with infants without IVH or AKI (*P* = 0.004) (Table 4).

**Table 4:** Comparison of demographic, clinical, and maternal characteristics among infants with and without IVH and/or AKI after matching.

Parameters	IVH with AKI (n = 99)	IVH without AKI (n = 99)	(*P* value)	IVH and KI (n = 94)	NO IVH with No AKI (n = 94)	(*P* value)
Gestational age, weeks	27 (26–30)	27 (26–29)	0.54	28 (26–30)	27 (26–29)	0.25
Birth weight, grams	950 (780–1145)	940 (775–1140)	0.87	982.5 (800–1166.25)	960 (784.75–1171.25)	0.88
Booked	15 (15.2)	14 (14.1)	> 0.99	13 (13.8)	10 (10.6)	0.65
Antenatal steroid treatment	49 (49.5)	48 (48.5)	> 0.99	42 (44.7)	56 (59.6)	0.05
Cesarean section delivery	46 (46.5)	41 (41.4)	0.56	40 (42.6)	38 (40.4)	0.88
Gestational diabetes mellitus	3 (3)	3 (3)	> 0.99	4 (4.3)	3 (3.2)	> 0.99
Maternal hypertension	17 (17.2)	24 (24.2)	0.29	17 (18.1)	23 (24.5)	0.37
1-min Apgar score	4 (2–6)	4 (3–6)	0.46	5 (2–6)	5 (4–6)	0.002*
5-min Apgar score	7 (6–7)	7 (6–7)	0.4	7 (6–8)	7 (6–8)	0.06
RDS surfactant use	83 (83.8)	76 (76.8)	0.28	77 (81.9)	73 (77.7)	0.58
Male sex	52 (52.5)	55 (55.6)	0.77	48 (51.1)	46 (48.9)	0.88
Non-invasive ventilator	76 (76.8)	80 (80.8)	0.6	77 (81.9)	80 (85.1)	0.69
Invasive ventilator	86 (86.9)	88 (88.9)	0.82	80 (85.1)	77 (81.9)	0.69
PDA medical treatment	75 (75.8)	63 (63.6)	0.08	70 (74.5)	62 (66)	0.26
Pneumothorax	13 (13.1)	7 (7.1)	0.23	13 (13.8)	5 (5.3)	0.08
Pulmonary hemorrhage	16 (16.2)	9 (9.1)	0.19	18 (19.1)	4 (4.3)	0.002*
EBM	50 (50.5)	52 (52.5)	0.88	48 (51.1)	48 (51.1)	> 0.99
Inotropes	69 (69.7)	54 (54.5)	0.04*	63 (67)	31 (33)	< 0.001*
Nephrotoxic medication^a^	96 (97)	94 (94.9)	0.72	90 (95.7)	88 (93.6)	0.74
UAC	46 (46.5)	48 (48.5)	0.88	41 (43.6)	41 (43.6)	> 0.99
UVC	86 (86.9)	86 (86.9)	> 0.99	80 (85.1)	85 (90.4)	0.37
EOS	2 (2.3)	4 (4.8)	0.44	2 (2.4)	1 (1.2)	> 0.99
LOS	36 (41.9)	33 (39.3)	0.75	35 (42.7)	26 (32.1)	0.19
LOHS	44 (26–74)	55 (27–87)	0.58	41 (17–78)	64.5 (25.25–95.25)	0.21
Mortality	40 (40.4)	23 (23.2)	0.01*	32 (34)	14 (14.9)	0.004*

Data are presented as median (IQR), or number (%), as appropriate.

Abbreviations: *IVH* intraventricular hemorrhage, *AKI* acute kidney injury, *GDM* gestational diabetes mellitus, *RDS* respiratory distress syndrome, *PDA* patent ductus arteriosus, *EBM* expressed breast milk, *UAC* umbilical arterial catheter, *UVC* umbilical venous catheter, *EOS* early-onset sepsis, *LOS* late-onset sepsis, *LOHS* length of hospital stay.

*Statistically significant at 5% level.

Bold values indicate all values less than 0.05 are significant.

^a^Nephrotoxic medication: Acyclovir, Amphotericin B, Aminoglycosides, Vancomycin.

The multivariable relative risk regression analysis was performed to predict the impact of independent significant risk factors including GA, BW, 1- and 5-min Apgar scores, surfactant use, and pulmonary hemorrhage, on the mortality of infants with IVH and AKI versus those with IVH but without AKI. The adjusted model showed that mortality was increased 1.5 times in infants with IVH and AKI (Table 5).

**Table 5:** Multivariable analysis of neonatal mortality stratified by IVH with AKI versus IVH without AKI and by IVH with AKI versus no IVH or AKI.

	IVH with AKI versus IVH without AKI						IVH with AKI versus no IVH and no AKI					
	Crude RR			Adjusted RR			Crude RR			Adjusted RR		
Outcome	RR	CI	(*P* value)	aRR	CI	(*P* value)	RR	CI	(*P* value)	aRR	CI	(*P* value)
Mortality	1.73	1.13–2.67	0.01*	1.51	1.02–2.24	0.03*	2.28	1.3–3.99	**0.004**	1.86	1.04–3.31	0.03*

Abbreviations: *IVH* intraventricular hemorrhage, *AKI* acute kidney injury, *RR* relative risk, *CI* confidence interval.

*Statistically significant at 5% level.

Bold values indicate all values less than 0.05 are significant.

Furthermore, after adjusting the relative risk of several independent significant risk factors (GA, BW, 1- and 5-min Apgar scores, pulmonary hemorrhage, expressed breast milk use, and UAC insertion), we found an association between IVH with AKI and an increased risk of mortality versus no IVH or AKI. (Table 5).

## Discussion

This study investigated the relationship between IVH and AKI in premature infants. Our results showed that premature infants with IVH were at risk of developing AKI; the risk was almost twice as high in the first two weeks of life compared with those who did not develop IVH within the same time period in the study. Furthermore, we found an association between having both IVH and AKI and increased risk of mortality within the hospital stay compared with those who developed IVH without AKI or those who did not develop IVH or AKI, even after controlling for the most important risk factors that can affect the outcome.

The origin of IVH is at the germinal matrix, which is a highly fragile, cellular, and gelatinous structure, and it is vascularized mainly by the terminal branches of the anterior cerebral artery, middle cerebral artery, and internal carotid artery. These branches contribute, through venous terminals, to the formation of a rich capillary network with free communication^16,17^. Therefore, cerebral blood flow is directly vulnerable to changes in systemic blood pressure. Intraventricular hemorrhage is multifactorial, and episodes of both systemic hypotension and hypertension can produce germinal matrix bleeding^18–20^. Premature infants who are younger than 32 weeks are easily exposed to perturbations in their arterial and venous circulations in the first week of life, which, in turn, increase the possibility of developing intraventricular hemorrhage^21^. Interestingly, initiation of IVH can cause metabolic acidosis, hypotension, bradycardia, temperature instability, and abnormal glucose metabolism^11^, which can compromise renal perfusion and cause AKI, especially in premature infants.

The Assessment of the Worldwide Acute Kidney Injury Epidemiology in Neonates (AWAKEN) study is a large multicenter retrospective study that investigated the incidence and outcomes of neonatal AKI^6^. The same researchers conducted another single-center cohort study to investigate the same hypothesis^22^, and their results showed that premature infants with AKI were more likely to have IVH, especially in those with a GA below 28 weeks^14^. Moreover, another recent retrospective study followed the same theory of the AWAKEN study and added fluid balance in its analysis^15^. They reached the same conclusion; i.e., that AKI increased the risk of IVH in the first week of life; however, it is a small study compared with the AWAKEN study and our study.

In the present study, we employed the same definition of AKI that was used in the AWAKEN study. The results of our study are similar with regard to the presence of a direct relationship between IVH and AKI. However, our results showed that IVH is an independent risk factor for developing AKI in premature infants. Regarding the common timing of IVH and AKI occurrence, a systematic review and meta-analysis found that the majority of IVH events occur within the first 6 h of life^23^. In the AWAKEN study, the investigators assumed that AKI could alter blood pressure regulation by changing the RAAS or by activating a systemic inflammatory cascade. In the AWAKEN study and in the present study, infants with congenital kidney and heart disease were excluded; thus, it was rare to find these mechanisms progressing in the first hours or even days of life to cause AKI, which, in turn, would cause IVH. One of the disadvantages of the Kidney Disease: Improving Global Outcomes (KDIGO) criteria for AKI diagnosis is that it takes at least 48–72 h to establish the diagnosis; furthermore, high creatinine levels in the first few days of neonatal life mostly reflect maternal creatinine concentrations^24,25^.

The strengths of our study are as follows: First, we compared IVH patients to examine whether the severity of IVH plays a role in the development of AKI. We found that patients with severe IVH (grades III and IV) developed AKI more often than those who had mild IVH (grades I and II). We matched GA and BW among the study groups to determine whether the association between IVH and AKI influences the mortality rate. Specifically, we found that the mortality rate was higher in premature infants who developed IVH and AKI together.

This study has some limitations. First, we did not consider the urine output (UOP) in our study because it is difficult to measure in premature infants. As our study is retrospective in nature, it was difficult to define some variables such as boluses in the first few days of life and the hemodynamic status of patent ductus arteriosus, as not all patients underwent an echocardiogram. However, we were able to control some variables that were not controlled in other studies, such as the rate of antenatal steroids; further, we matched the GA and BW among the premature infants. The head ultrasound images were reviewed and reported by a pediatric radiologist who was not aware of the study subject. As many infants were included, we stratified the outcome according to GA. In the future, a large prospective study that investigates the timing of IVH occurrence using serial head ultrasound, non-invasive observation of cerebral and renal blood flow using near-infrared spectroscopy technology, and daily serum creatinine and UOP observation should be performed.

In conclusion, there is a significant association between IVH and AKI in premature infants; this association might increase the incidence of mortality in premature infants. To the best of our knowledge, this is the first study to investigate the influence of the association between IVH and AKI on the neonatal mortality rate.

## Methods

### Study design

In this retrospective cohort study, the charts of premature infants (GA ≤ 32 weeks; BW < 1500 g) who were admitted to the NICU of King Saud Medical City (KSMC; a tertiary referral center) from July 2014 to December 2020 were reviewed.

The NICU has an average annual admission of 1100 patients and includes NICU levels 2 and 3. This study was conducted in accordance with the Declaration of Helsinki and the Good Pharmacoepidemiology Practices guidelines and was approved by the Medical Ethical Review Committee of KSMC (reference number: H1RI-21-Dec17-01), which also waived the requirement for consent.

### Definitions

IVH was classified into grades I–IV according to the IVH classification described by Papile et al.^26^. IVH was diagnosed based on the findings of a head ultrasound performed between days 5 and 7 after birth^26,27^. All ultrasound findings were reviewed by an expert pediatric radiologist, and 7.5- and 10-MHz transducers (LOGIQ e; GE Medical Systems Co., Ltd., Jiangsu, China) were used to perform the ultrasound in the sagittal and coronal planes.

AKI was defined according to the neonate modified KDIGO criteria based on SCr and/or UOP criteria^28^. The UOP criteria were not included because measurement of UOP in premature infants is often difficult, and non-oliguric AKI is very common in this population. Baseline SCr was defined as the lowest previous value. The AKI stages were defined as follows: stage 1, increase in SCr ≥ 0.3 mg/dL within 48 h or ≥ 150% of baseline within 7 days; stage 2, a 200% increase in SCr from baseline; stage 3, an increase in SCr ≥ 2.5 mg/dL, ≥ 300% increase in SCr from baseline, or undergoing dialysis.

### Inclusion and exclusion criteria

Infants born at the KSMC at ≤ 32 gestational weeks with a BW < 1500 g who were admitted to the NICU were included in this study. Infants without IVH or AKI were assigned to the control group. Infants were categorized into two groups according to GA: < 28 and 28–32 gestational weeks.

Infants with a known congenital abnormality of the kidney or urinary tract, those with major congenital anomalies, those who were not born at KSMC, those who had a congenital infection, those whose mother had nephrotic syndrome, or those for whom data could not be retrieved were excluded from the study.

A sample size of 710 premature infants was analyzed to detect differences in the short-term outcome.

### Data collection and follow-up

Patients’ charts from NICU admission until discharge or death were reviewed. Demographic, clinical, and outcome data of all infants were obtained. Maternal data, including the presence of gestational diabetes mellitus, maternal hypertension, antenatal steroid treatment, and mode of delivery, were also obtained.

### Study outcomes

The primary outcome of this study was to investigate the relationship between IVH and AKI in premature infants and to determine whether this association affects the incidence of neonatal mortality.

### Statistical analysis

Before beginning the analysis, the data set was checked for missing data. Data were analyzed using the Statistical Package for the Social Sciences version 25.0 (IBM Corp., Armonk, NY, USA). Descriptive statistics, including the median, interquartile range, frequency, and percentages, were used to describe the maternal and neonatal variables. Fisher’s exact test was used to determine the association between categorical variables. Further, the Mann–Whitney U test was used for ordinal qualitative variables (GA, BW, and Apgar scores). For continuous variables, the unpaired Student *t*-test was used; for non-normally distributed data, the Mann–Whitney U test was used. The Kolmogorov–Smirnov test and a visual inspection of histograms were performed to verify the normality of the distribution of the quantitative variables. The association between IVH grades and AKI stages was analyzed using the Cochran–Mantel–Haenszel test. An ordinal logistic regression model was used to estimate the odds ratios and associated 95% confidence intervals for the association between IVH and AKI. All factors with a *P* value < 0.05 in the univariable analysis were considered for inclusion in the final multivariable ordinal regression model. Modified log-Poisson regression in generalized linear models with robust variance estimator (Huber–White) was applied for univariable relative risk analysis and to the models to adjust the relative risk of the infants’ mortality outcomes. Possible interactions between IVH and AKI were also examined. If the interaction was not significant, it was not included in the model. Independent variables in the models were selected from variables that were significantly related to death in the univariable analysis. All statistical tests were two-tailed, and *P* values < 0.05 were considered statistically significant.

## Data Availability

The data sets analyzed during this study are available from the corresponding author on reasonable request.

## References

[1] Stoll BJ (2010). Neonatal outcomes of extremely preterm infants from the NICHD Neonatal Research Network. Pediatrics.

[2] Stoll BJ (2015). Trends in care practices, morbidity, and mortality of extremely preterm neonates, 1993–2012. JAMA.

[3] O'Shea TM (2012). Intraventricular hemorrhage and developmental outcomes at 24 months of age in extremely preterm infants. J. Child. Neurol..

[4] Bolisetty S (2014). Intraventricular hemorrhage and neurodevelopmental outcomes in extreme preterm infants. Pediatrics.

[5] Liu M, Chien CC, Grigoryev DN, Gandolfo MT, Colvin RB, Rabb H (2009). Effect of T cells on vascular permeability in early ischemic acute kidney injury in mice. Microvasc. Res..

[6] Koralkar R, Ambalavanan N, Levitan EB, McGwin G, Goldstein S, Askenazi D (2011). Acute kidney injury reduces survival in very low birth weight infants. Pediatr. Res..

[7] Carmody JB, Swanson JR, Rhone ET, Charlton JR (2014). Recognition and reporting of AKI in very low birth weight infants. Clin. J. Am. Soc. Nephrol..

[8] Selewski DT (2015). Neonatal acute kidney injury. Pediatrics.

[9] Jetton JG (2017). Incidence and outcomes of neonatal acute kidney injury (AWAKEN): A multicentre, multinational, observational cohort study. Lancet Child Adolesc. Health.

[10] Askenazi DJ (2013). Fluid overload and mortality are associated with acute kidney injury in sick near-term/term neonate. Pediatr. Nephrol..

[11] Volpe JJ (2017). Volpe's Neurology of the Newborn E-Book.

[12] Volpe JJ (2008). Neurology of the Newborn E-Book.

[13] Mahieu-Caputo D (2000). Twin-to-twin transfusion syndrome. Role of the fetal renin–angiotensin system. Am. J. Pathol..

[14] Stoops C (2019). The association of intraventricular hemorrhage and acute kidney injury in premature infants from the Assessment of the Worldwide Acute Kidney Injury Epidemiology in Neonates (AWAKEN) study. Neonatology.

[15] Adcock B (2020). Acute kidney injury, fluid balance and risks of intraventricular hemorrhage in premature infants. J. Perinatol..

[16] Hambleton G, Wigglesworth JS (1976). Origin of intraventricular haemorrhage in the preterm infant. Arch. Dis. Child..

[17] Takashima S, Tanaka K (1978). Microangiography and vascular permeability of the subependymal matrix in the premature infant. Can. J. Neurol. Sci..

[18] Goddard-Finegold J, Armstrong D, Zeller RS (1982). Intraventricular hemorrhage, following volume expansion after hypovolemic hypotension in the newborn beagle. J. Pediatr..

[19] Ment LR, Stewart WB, Duncan CC, Lambrecht R (1982). Beagle puppy model of intraventricular hemorrhage. J. Neurosurg..

[20] Al-Mouqdad, M. M. *et al*. Risk factors for intraventricular hemorrhage in premature infants in the central region of Saudi Arabia. *Int J Pediatr Adolesc Med.* **8**(2), 76–81. 10.1016/j.ijpam.2019.11.005 (2019). 10.1016/j.ijpam.2019.11.005 PMC8144857 34084876

[21] de Vries LS, van Rijn AMR, Rademaker KJ, Van Haastert IC, Beek FJ, Groenendaal F (2001). Unilateral parenchymal haemorrhagic infarction in the preterm infant. Eur. J. Paediatr. Neurol..

[22] Stoops C, Sims B, Griffin R, Askenazi DJ (2016). Neonatal acute kidney injury and the risk of intraventricular hemorrhage in the very low birth weight infant. Neonatology.

[23] Al-Abdi SY, Al-Aamri MA (2014). A systematic review and meta-analysis of the timing of early intraventricular hemorrhage in preterm neonates: Clinical and research implications. J. Clin. Neonatol..

[24] Gordjani N, Burghard R, Leititis JU, Brandis M (1988). Serum creatinine and creatinine clearance in healthy neonates and prematures during the first 10 days of life. Eur. J. Pediatr..

[25] Wilkins BH (1992). Renal function in sick very low birthweight infants: 3. Sodium, potassium, and water excretion. Arch. Dis. Child..

[26] Ballabh P (2010). Intraventricular hemorrhage in premature infants: Mechanism of disease. Pediatr. Res..

[27] Huvanandana J, Nguyen C, Thamrin C, Tracy M, Hinder M, McEwan AL (2017). Prediction of intraventricular haemorrhage in preterm infants using time series analysis of blood pressure and respiratory signals. Sci. Rep..

[28] Jetton JG, Askenazi DJ (2012). Update on acute kidney injury in the neonate. Curr. Opin. Pediatr..

